# Spiking Neurons in a Hierarchical Self-Organizing Map Model Can Learn to Develop Spatial and Temporal Properties of Entorhinal Grid Cells and Hippocampal Place Cells

**DOI:** 10.1371/journal.pone.0060599

**Published:** 2013-04-05

**Authors:** Praveen K. Pilly, Stephen Grossberg

**Affiliations:** 1 Center for Adaptive Systems, Center for Computational Neuroscience and Neural Technology, Boston University, Boston, Massachusetts, United States of America; 2 Center for Adaptive Systems, Center for Computational Neuroscience and Neural Technology, Department of Mathematics, Boston University, Boston, Massachusetts, United States of America; SUNY Downstate MC, United States of America

## Abstract

Medial entorhinal grid cells and hippocampal place cells provide neural correlates of spatial representation in the brain. A place cell typically fires whenever an animal is present in one or more spatial regions, or places, of an environment. A grid cell typically fires in multiple spatial regions that form a regular hexagonal grid structure extending throughout the environment. Different grid and place cells prefer spatially offset regions, with their firing fields increasing in size along the dorsoventral axes of the medial entorhinal cortex and hippocampus. The spacing between neighboring fields for a grid cell also increases along the dorsoventral axis. This article presents a neural model whose spiking neurons operate in a hierarchy of self-organizing maps, each obeying the same laws. This spiking GridPlaceMap model simulates how grid cells and place cells may develop. It responds to realistic rat navigational trajectories by learning grid cells with hexagonal grid firing fields of multiple spatial scales and place cells with one or more firing fields that match neurophysiological data about these cells and their development in juvenile rats. The place cells represent much larger spaces than the grid cells, which enable them to support navigational behaviors. Both self-organizing maps amplify and learn to categorize the most frequent and energetic co-occurrences of their inputs. The current results build upon a previous rate-based model of grid and place cell learning, and thus illustrate a general method for converting rate-based adaptive neural models, without the loss of any of their analog properties, into models whose cells obey spiking dynamics. New properties of the spiking GridPlaceMap model include the appearance of theta band modulation. The spiking model also opens a path for implementation in brain-emulating nanochips comprised of networks of noisy spiking neurons with multiple-level adaptive weights for controlling autonomous adaptive robots capable of spatial navigation.

## Introduction

How our brains acquire stable cognitive maps of the spatial environments that we explore is not only an outstanding scientific question, but also one with immense potential for technological applications. For example, this knowledge can be applied in designing autonomous agents that are capable of spatial cognition and navigation in a GPS signal-impoverished environment without the need for human teleoperation.

Lesion and pharmacological studies have revealed that hippocampus (HC) and medial entorhinal cortex (MEC) are critical brain areas for spatial learning, memory, and behavior [1]–[3]. Place cells in HC fire whenever the rat is positioned in a specific localized region, or “place”, of an environment [4]. Place cells have also been observed to exhibit multiple firing fields in large spaces [5]–[7]. Different place cells prefer different regions, and the place cell ensemble code enables the animal to localize itself in an environment. Remarkably, grid cells in superficial layers of MEC fire in multiple places that may form a regular hexagonal grid across the navigable environment [8]. It should be noted that although place cells can have multiple fields in a large space, they do not exhibit any noticeable spatial periodicity in their responses [5], [7].

Since the time of the proposal of [9], research on place cells has disclosed that they receive two kinds of inputs: one conveying information about the sensory context experienced from a given place, and the other from a navigational, or path integration, system, which tracks relative position in the world by integrating self-movement angular and linear velocity estimates for instantaneous rotation and translation, respectively; see below. An important open problem is to explain how sensory context and path integration information are combined in the control of navigation.

Sensory context includes properties of the following kind: [10] demonstrated that place cells active in a walled enclosure show selectivity to the distances of the preferred place from the wall in various directions. [11] modeled the learning of place fields for cells receiving adaptive inputs from hypothetical boundary vector cells [12], which fire preferentially to the presence of a boundary (e.g., wall, sheer drop) at a particular distance in a particular world-centered direction. [13] reported that about 24% of subicular cells have properties similar to those of predicted boundary vector cells, even though most of these cells had tuning to only shorter distances.

The primary determinants of grid cell firing are, however, path integration-based inputs [14]. Indeed, the environmental signals sensed at each of the various hexagonally-distributed spatial firing positions of a single grid cell are different. Being one synapse upstream of hippocampal CA1 and CA3 place cells, the ensemble of entorhinal grid cells may represent the main processed output of the path integration system. The spacing between neighboring fields and the field sizes of grid cells increase, on average, from the dorsal to the ventral end of the MEC [15]–[17]. Moreover, the spatial fields of grid cells recorded from a given dorsovental location in rat MEC exhibit different phases; i.e., they are offset from each other [8]. These properties led to the suggestion that a place cell with spatial selectivity for a given position can be derived by selectively combining grid cells with multiple spatial phases and scales that are co-active at that position, in such a way that the grid-to-place transformation allows for the expansion of the scale of spatial representation in the brain [14], [18]. In other words, the maximal size of the environment in which a place cell exhibits only a single firing field can be much larger than the individual spatial scales of grid cells that are combined to fire the place cell. Some self-organizing implementations of this concept have been proposed in which place fields in one-dimensional and two-dimensional spaces are learned based on inputs from hard-wired grid cells of multiple spatial scales and phases [19]–[22].

Along similar lines, [23] proposed the GRIDSmap model to show that grid cells can themselves be self-organized as spatial categories in response to inputs from hypothesized stripe cells whose function is to integrate linear velocity inputs. Just as head direction (HD) cells [24], [25] have been conceptualized to integrate angular head velocity signals using a ring attractor circuit (e.g., [26]–[28]), stripe cells were proposed to employ the same neural design for linear velocity path integration. HD cells and stripe cells are arranged in a ring within these circuits, and are activated as the activity bump that represents integrated angular or linear velocity signals passes over their positions in the ring; hence the name “ring attractor” for this type of model. While only one ring attractor is sufficient to model HD cells, several stripe cell ring attractors are needed for integrating linear velocity along different directions (i.e., not just forward and backward) and over different finite spacings. The firing of stripe cells can thus be characterized by four parameters; namely, stripe spacing, stripe field width, spatial phase, and preferred direction. Stripe cells are so named because their spatial firing patterns resemble parallel stripes that cover the entire environment. The rate at which the activity bump of a stripe cell ring attractor completes one revolution in response to translational movement with a component along its preferred direction is inversely proportional to the spacing of the constituent stripe cells.

Why do grid cells learn to fire at hexagonally-located positions as an animal navigates in an open field? [23] and [29] showed, using simple trigonometry-based analysis, that self-organizing entorhinal map cells are more likely to learn hexagonal grid fields because, among all possible input combinations of stripe cells with the same spacing, the ones that are most frequently and energetically co-activated are sets consisting of three co-active stripe cells whose preferred directions differ from each other by 60°, and these preferred stripe cell sets are activated at positions that form a regular hexagonal grid across two-dimensional space. The **Discussion** section reviews how hexagonal grid structures can be learned in the brain even when stripe cells of multiple spacings converge initially on entorhinal cells [30]. The predicted existence of stripe cells has recently received experimental support from a report of cells with such spatial firing properties in dorsal parasubiculum [31], which projects directly to layer II of medial entorhinal cortex [32], [33].

Most computational models focused on learning of either hippocampal place cells [19]–[22] or entorhinal grid cells [23]. [29] were the first to model how both grid and place cells, despite the different appearances of their receptive fields, can emerge during development using the same network and synaptic laws. In particular, they presented the unified GridPlaceMap model to demonstrate that a hierarchy of self-organizing maps (SOMs), each obeying the same laws, can concurrently learn characteristic grid fields and place fields at its first and second stages, respectively, in response to inputs from stripe cells. This occurs as a natural result of how self-organizing map cells at either stage gradually develop selectivities, or categories, for the most frequent and energetic coactivation patterns occurring in their respective input streams. The GridPlaceMap model is also able to quantitatively simulate neurophysiological data from rat pups regarding the development of grid and place cells during the third and fourth weeks after birth (P15-P28) when they begin to explore their environments for the first time [34], [35]. Further, with regard to grid cell learning, GridPlaceMap goes beyond the GRIDSmap model by refining the explanation for the self-organized emergence of hexagonal grid fields; and identifying minimal and necessary mechanisms to learn grid fields with a higher hexagonal gridness quality, in a larger population of map cells, and in response to a greater variation in stripe cell parameters. The assumption of developed, or perhaps hard-wired, stripe cells to drive spatial learning in the entorhinal-hippocampal system is consistent with the existence of adultlike HD cells in the parahippocampal regions of juvenile rats already by P14 [34], [35], when spatial exploration first begins.

The original GridPlaceMap model uses neurons that interact using rate coding; that is, they interact via signals based on spiking frequency, rather than in terms of their individual spike trains. The goals of the current model are threefold; namely, to test whether the insights gained from the rate-based GridPlaceMap model can be applied and extended to simulating and explaining the development of spiking grid and place cells, as an instantiation of a general method for converting rate-based adaptive neural models, without the loss of any of their analog properties, into models whose cells obey spiking dynamics; to develop a neural system that makes it possible to address, for the first time, known temporal coding properties of hippocampal place cells and medial entorhinal layer II grid cells, such as theta band modulation [34], [35], as emergent properties of network interactions that support grid and place cell learning; and to contribute towards building a spiking implementation, in low-power high-density neuromorphic hardware, of an architecture for spatial navigation, goal-oriented search, and cognitive planning in future biologically-inspired autonomous mobile robots.

Additional extensions of the GridPlaceMap and sGridPlaceMap models will be needed to achieve a general-purpose neural architecture for spatial navigation. It has, for example, been proposed how top-down attentive matching processes from hippocampal to entorhinal cortex may facilitate fast learning and dynamic self-stabilization of learned spatial memories, provide a pathway whereby environmental cues may modulate properties of grid and place cells that arise through path integration, and may help to explain a wide range of additional data about modular grid orientations, grid realignment, place remapping, and gamma and beta oscillations (e.g., [29], [36]).

## Methods

**Figure 1:**
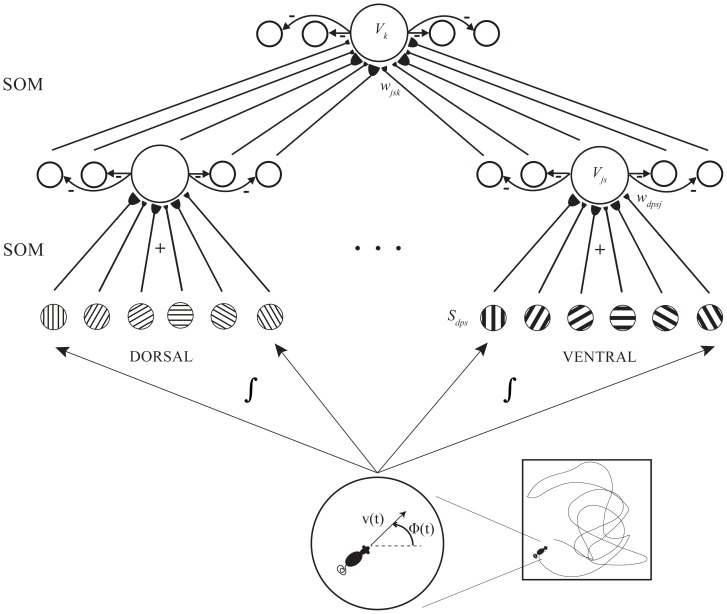
sGridPlaceMap model diagram. sGridPlaceMap demonstrates the hierarchical self-organization of spiking grid cells of multiple spatial scales and of spiking place cells in response to path integration-based inputs. Model simulations were conducted with 100 hippocampal map cells, three populations comprising 100 map cells each at different locations along the dorsoventral axis of medial entorhinal cortex, and stripe cells with three spacings, 18 direction preferences, and five spatial phases. [Figure reprinted with permission from [29].].

The spiking GridPlaceMap model, called sGridPlaceMap (see Figure 1), employs leaky integrate-and-fire neurons [37] whose membrane potential dynamics are controlled by synaptic currents mediated by NMDA and GABA_A_ receptors, and whose synaptic plasticity is governed by a spike timing-dependent variant of the competitive instar learning law [38], [39]. This is the first application of spike-triggered competitive instar learning. Analog activity dependence of the learned adaptive weights is realized by temporally leaky trace variables that are reset to their full value of one by spiking in the corresponding pre-synaptic neurons. Self-normalized weights are learned due to competition among synaptic sites as per the competitive instar learning law, which is experimentally supported by data on the competition among developing axons abutting a target neuron for limited target-derived neurotrophic factor support in order to survive [40]–[42], and the conservation of total synaptic weight [43].

Since the focus of the present study is to show how spiking dynamics can drive learning of grid and place cell receptive fields, with an eye towards implementation in neuromorphic hardware, rather than fidelity to all biophysical subtleties, each neuron is represented by a single compartment, which lumps together the soma and its dendritic elements. In addition, voltage-gated fast Na^+^ channels and delayed rectifier K^+^ channels that underlie the generation of stereotypical spike waveforms in membrane potential dynamics, synaptic transmission delays, axonal conduction latencies, and refractory periods are not considered. GABA_A_-gated channel conductances are approximated by single exponentially decaying traces because their rise times are typically negligible (e.g., [44]). If a pre-synaptic spike arrives at the synaptic cleft before the inhibitory ion channel closes, then its conductance is, nonetheless, reset to its fully open state (i.e., maximal value). NMDA-gated channel conductances are modeled using two multiplicative terms, one that incorporates sensitivity to postsynaptic membrane depolarization and the other that accounts for glutamate binding kinetics. AMPA-gated channels, which regulate the fast components of excitatory postsynaptic potentials (EPSPs), are not explicitly included because there are no clear data on the NMDA/AMPA receptor density ratios for entorhinal stellate cells and hippocampal pyramidal cells before postnatal development of the spatial representation maps begins. NMDA receptors are included because they are widely accepted to be relatively more indispensable to long-term potentiation in general (e.g., [45], [46]) and to spatial learning and memory in particular (e.g., [47]). Further, the slow dynamics of NMDA receptor-mediated EPSPs allow greater temporal summation of spikes from input neurons that are not precisely coincident.

Our results suggest that this granularity of neuronal modeling, which is at a finer level compared to GridPlaceMap simulations, is sufficient for the purposes of studying the development of functional spiking grid and place cells, and also minimal enough for very large-scale incorporation in neuromorphic hardware. MATLAB code to implement the model is available at the following link: https://senselab.med.yale.edu/modeldb/ShowModel.asp?model=148035.

### sGridPlaceMap model description

#### Stripe cells

As noted above, stripe cells for linear path integration and head direction cells for angular path integration are both proposed to be realized by ring attractor circuits. Several authors have earlier proposed that head direction cells may be modeled as ring attractors in which angular head velocity signals are integrated through time into displacements of an activity bump along the ring [26]–[28]. In like manner, the GRIDSmap, GridPlaceMap, and spiking GridPlaceMap models all assume that linear velocity along different prescribed directions are integrated in different ring attractors into displacements of activity bumps along the corresponding rings. Stripe cells are the individual cells within each such ring attractor circuit and are thus activated at different spatial phases as the activity bump moves across their ring locations. They may be activated periodically as the activity bump moves around the ring attractor more than once in response to the navigational movements of the animal. The outputs of head direction cells modulate the linear velocity signal for driving the various directionally-selective stripe cell ring attractor circuits.

The stripe cell ring attractors are modeled algorithmically, for simplicity. They generate probabilistically determined spike trains to the self-organizing map hierarchy of spiking entorhinal and hippocampal cells in the following way.

Suppose that at time 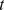 the animal is heading along allocentric direction 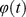 with linear velocity 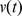. Then the velocity 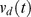 along direction 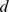 is:
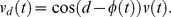
(1)


The displacement 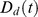 that is traveled along direction 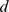 with respect to the initial position is calculated by integrating the corresponding velocity:
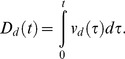
(2)


This directional displacement variable is converted into activities of various stripe cells that prefer direction 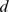. In particular, the firing rate 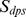 of the stripe cell with phase 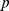 along a ring attractor corresponding to direction 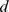
and spacing 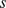 is maximal at periodic positions 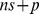 along its preferred direction, for all integer values of 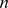. In other words, 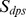 will be maximal whenever (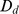 modulo 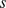) = 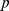. Defining the spatial phase difference 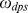 between 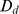 and 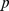 with respect to the orbit of the activity bump for the corresponding ring attractor by:
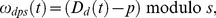
(3) the firing rate 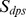 of the stripe cell is then modeled by a Gaussian tuning function: 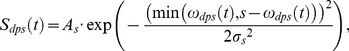
(4) where 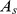 is the peak firing rate (in Hz) and 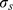 is the standard deviation describing the width of each of its individual stripe fields along preferred direction 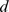.

All directional displacement variables 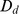 are initialized to 0 at the start of each learning trial. The navigated trajectory hereby determines the firing rates of all stripe cells via Equations 1–4, which in turn control the generation of their non-homogenous Poisson spike trains using the method of infinitesimal increments. Briefly, a cell with an instantaneous firing rate of 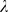 fires a spike within an infinitesimal duration (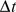) if 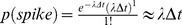 is greater than a random number sampled from a uniform distribution between 0 and 1.

The remainder of the model description describes the SOM equations for the development of entorhinal grid cells (Equations 5–11) and hippocampal place cells (Equations 12–18):

#### Medial entorhinal cortex (MEC) map cells

The membrane potential 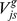 of the 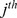 MEC map cell of scale 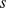 is defined by a membrane equation that obeys shunting integrate-and-fire dynamics within a recurrent competitive network:
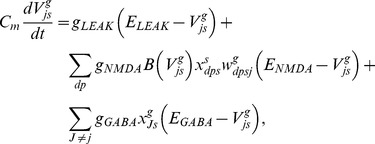
(5) where 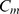 is membrane capacitance; 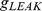 is the constant conductance of the leak Cl^−^ channel; 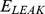 is the reversal potential of the leak Cl^−^ channel; 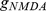 is the maximal conductance of each excitatory NMDA receptor-mediated channel; 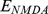 is the corresponding reversal potential; 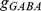 is the maximal conductance of each inhibitory, GABA_A_ receptor-mediated channel; 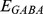 is the corresponding reversal potential; 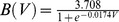 defines the voltage-dependent removal of the Mg^2+^ block in the NMDA channel [48]; 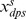 is the NMDA channel gating variable that is controlled by the spiking of the stripe cell that codes direction 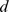, phase 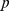, and scale 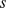; 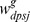 is the synaptic weight of the projection from this stripe cell to the 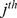 MEC map cell of scale 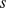; and 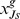 is the GABA_A_ channel conductance gate, modeled as a single exponential wave, that is opened by the spiking of the 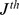 MEC map cell of scale 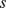 in the off-surround. The dynamics of the NMDA channel gating variable 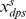 obey a mass action law [49]:
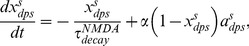
(6) where the secondary gating variable 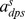 obeys:
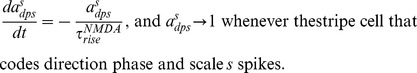
(7)


The secondary gates 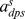 may be interpreted as AMPA channels, which help to kick start the activation of NMDA channels. Consistent with this view, the value of the time constant 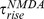 is relatively short similar to the typically reported time constants of AMPA channels. All gates are initialized to zero, and all membrane potentials are initialized to 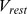 at the start of each learning trial. Whenever the membrane potential 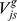 reaches the spiking threshold 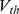, it is reset to 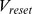, and the map cell triggers an output spike. The dynamics of the GABA_A_ channel conductance gate 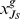 obey:
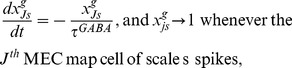
(8)


The adaptive weights, 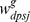, of the synaptic connections from stripe cells to MEC cells are modified using a spike timing-dependent variant of the competitive instar learning law, as follows:
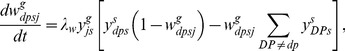
(9) where 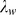 scales the rate of learning; 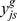 is a learning gate that is opened transiently by the spiking of the post-synaptic map cell 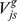; and 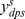 is an exponentially decaying trace variable that tracks the spiking activity of the stripe cell that codes direction 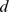, phase 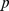, and scale 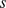. The learning gate 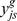 and the trace variable 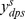 may be interpreted as a transient [Ca^2+^] increase in dendritic spines that is caused by a backpropagating action potential (bAP) via voltage-dependent Ca^2+^ channels, and an EPSP mediated by NMDA receptors, respectively [50]. Their dynamics obey:
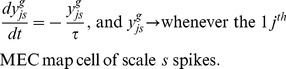
(10)

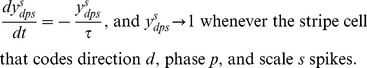
(11)


These variables are initialized to zero at the start of each trial. The weights are only initialized once, at the start of the first trial, by sampling from a uniform distribution between 0 and 0.1. The learning law in Equation 9 ensures that only a map cell that has recently spiked can trigger learning within its afferent synaptic weights; that is, learning can only occur when the gating signal 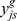 is positive. During this learning episode, each adaptive weight 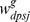 has a maximum value of 1 towards which its pre-synaptic input trace 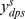 drives it, while all the other input traces 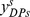 together compete against it as they attempt to augment their own weights. This cooperative-competitive process has the effect of normalizing the learned weights. In other words, the weights approach the ratio of the time-averaged inputs converging on the cell while the learning gate is open.

#### Hippocampal cortex (HC) maps cells

The membrane potential 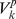 of the 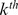 HC map cell is also governed by shunting integrate-and-fire dynamics within a recurrent competitive network:
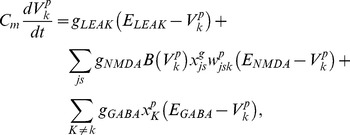
(12) where the parameters are the same as in Equation 5, 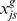 is the gating variable that is controlled by the spiking of the 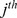 MEC map cell of scale 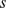; 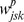 is the synaptic weight of the projection from this MEC map cell to the 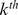 HC map cell; and 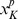 is the GABA_A_ channel conductance gate that is opened by the spiking of the 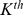 HC map cell in the off surround. As in Equation 5, the dynamics of the NMDA channel gating variable 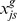 obey a mass action law [49]:
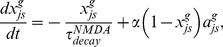
(13) where the secondary gating variable 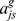 obeys:
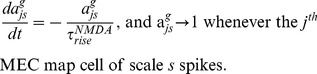
(14)


For this stage too, all gates are initialized to zero, and all membrane potentials are initialized to 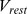 at the start of each trial. The dynamics of the GABA_A_ channel conductance gate 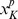 obey:
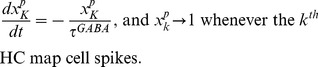
(15)


The adaptive weights, 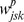, of the synaptic connections from MEC cells to HC cells are also modified using the spike timing-dependent competitive instar learning law, as follows:
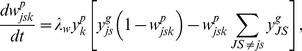
(16) where 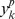 is a learning gate that is opened transiently by the spiking of the 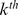 HC map cell; and 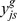 is an exponentially decaying trace variable that tracks the spiking activity of the 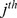 MEC map cell of scale 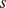. As in Equation 9, the dynamics of the learning gate 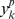 and trace variable 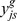 obey:
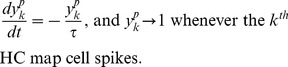
(17)

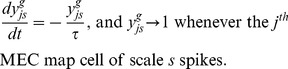
(18)


These variables are also initialized to zero at the start of each trial. The pre-learning weights are sampled from a uniform distribution between 0 and 0.03. The initial weights of projections from stripe cells to MEC cells have a higher individual mean to compensate for the relatively lower number of input cells; see below.

### Simulation settings

The parameter values used in the simulations were 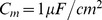; 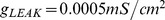; 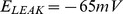; 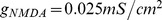; 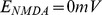; 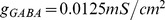; 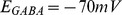; 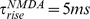; 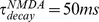; 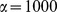; 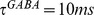; 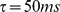; 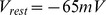; 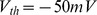; 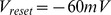; and 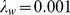. Note that the values for most of the parameters are the ones that are typically used in biophysically realistic simulations; namely, 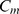, 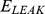, 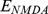, 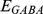, 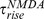, 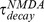; 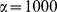, 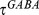, 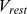, 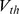, and 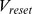. The differential equations, governing membrane potential and synaptic weight dynamics, were numerically integrated using Euler's forward method with a fixed time step 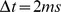. Input stripe cells were assumed with three spacings (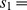
20 cm, 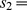
35 cm, and 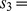
50 cm), 18 direction preferences (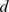:−90° to 80° in steps of 10°), and five spatial phases (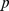 = [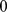, 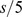, 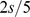, 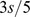, 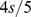] for the stripe spacing 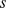) per direction. The values for the stripe spacings were chosen to match the observed constant ratio (1∶∼1.7∶∼2.5) of the smallest three grid spacings across rats [51]. The peak firing rate of stripe cells was assumed to be inversely proportional to stripe spacing, similar to how the peak rate of grid cells decreases with spatial scale [16]. In particular, the values used were 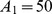 Hz, 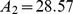 Hz, and 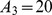 Hz. Stripe field width varied in proportion to stripe spacing, with the standard deviation 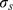 of each stripe field along its preferred direction set to 7% of the stripe spacing.

The model was run with 100 HC map cells receiving adaptive inputs from three distinct populations of 100 MEC map cells, each of which was driven by adaptive inputs from stripe cells of one of three spacings. Stripe cells were activated in response to linear velocity estimates derived from realistic rat trajectories of ∼10 min in a 100 cm×100 cm environment (primary data: [15]). 30 learning trials were employed, with each trial comprising one run of the animat across the environment. A novel trajectory was created for each trial by rotating the original rat trajectory by a random angle about the midpoint. In order to ensure that such derived trajectories go beyond the square environment only minimally, the original trajectory was prefixed by a short linear trajectory from the midpoint to the actual starting position at a running speed of 15 cm/s. The remaining minimal outer excursions were bounded by the environment's limits.

### Post-processing

The 100 cm×100 cm environment was divided into 2.5 cm×2.5 cm bins. During each trial, the amount of time spent by the animat in the various spatial bins was tracked. Also, for each map cell the number of spikes generated in the various bins was tracked. At the end of each trial, the resulting occupancy and spike count maps were smoothed using a 5×5 Gaussian kernel with standard deviation equal to one. Smoothed and unsmoothed spatial rate maps for each map cell were obtained by dividing the corresponding spike count variable by corresponding occupancy variable across the bins. For each MEC map cell, six local maxima with 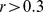 and closest to the central peak in the spatial autocorrelogram of its smoothed rate map were identified. Grid spacing was obtained as the median of their distances from the central peak [8], and grid score, which measures how hexagonal and periodic a grid pattern is, was computed using the method described in [35]. Grid orientation was defined as the smallest positive angle with the horizontal axis (0° direction) made by line segments connecting the central peak to each of these local maxima [8]. For each HC map cell, spatial information, which measures how predictive of the animal's spatial position a cell's firing rate is, was computed using adaptively smoothed rate maps [52], [53]. Inter-trial stability of a cell in a given trial was defined as the correlation coefficient between its smoothed rate maps from that trial and the immediately preceding one, considering only those bins with rate greater than zero in at least one of the trials [35]. Grid cells were defined as those MEC map cells whose grid score >0.3, and place cells as those HC map cells whose spatial information >0.5 [34], [35]. For each spatial scale, learned grid cells were clustered into different unique groups using the criterion that two grid firing patterns are similar if their spatial correlation coefficient 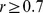 and their orientation difference 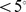 [29]. Similarly, learned place cells were grouped using the definition that two spatial firing patterns are similar if their spatial correlation coefficient 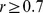 [29].

For each hippocampal cell, the spatial fields expressed over the course of a given trial were characterized with respect to their number, sizes, and nearest neighbor spacings (in case of multiple fields) from its adaptively smoothed firing rate map. Distinct fields were indentified from circular templates around local peaks based on the criteria that the maximal rate within a field is at least more than 50% of the overall peak rate [34], and the field has a minimum diameter of 3 bins (bin width  = 2.5 cm) with the average activity of the circumferential bins being equal to or less than 10% of the overall peak rate [16]. Further, if any pair of local peaks was connected by a straight segment of active bins whose activity was at least more than 20% of the overall peak rate, then the lower of the two peaks was not considered for the identification of distinct fields [54].

Temporal modulation in the spiking responses of cells was assessed by computing the power spectra of the corresponding spike trains, with a temporal resolution of 2 ms, using a standard procedure [34]. First, the autocorrelation of a given spike train is computed, which is truncated at a lag of 500 ms. Second, the signal is zero-mean normalized to remove the power at zero frequency. Third, it is tapered with a Hamming window to minimize spectral leakage. Finally, a discrete Fourier transform is applied (with 2^16^ points) whose amplitude response is squared, and normalized to the maximal value, to yield the power spectrum between 0 Hz and 250 Hz.

## Results

### Development of grid cells and place cells during spatial navigation

**Figure 2:**
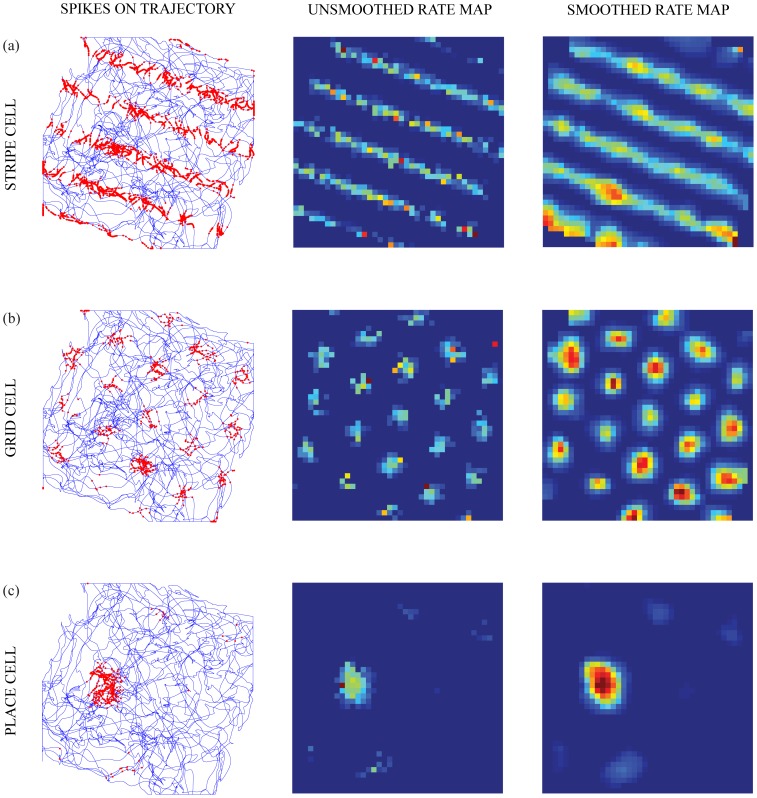
Spiking stripe, grid, and place cells. Spatial responses of representative (a) stripe, (b) grid, and (c) place cells. The first column shows the spike locations (red dots) of the cells superimposed on the trajectory of the animat during a trial. The second and third columns show the unsmoothed and smoothed spatial rate maps, respectively, of the cells. See **Methods** section for how spike recordings are converted into rate maps. Color coding from blue (min.) to red (max.) is used for each rate map.

This section shows that all the results of the rate-based GridPlaceMap model [29] are replicated by the spiking adaptive dynamics of sGridPlaceMap, in addition to accounting for theta band modulation and multiple place fields. Figure 2 illustrates model examples of spiking stripe, grid, and place cells during traversal of the animat along a realistic trajectory in two-dimensional space. The grid and place cell properties emerge through hierarchical self-organized learning. Table 1 summarizes the number and proportion of learned grid and place cells in the entorhinal and hippocampal maps, respectively. In particular, the model learned 78 unique grid fields (out of 100 map cells) for the input stripe spacing of 20 cm, 80 grid fields for 35 cm, 84 grid fields for 50 cm, and 56 unique place fields (out of 100 map cells).

**Table 1:** Tabulation of the learned grid and place cells.

(in last trial)	
No. of grid cells (20 cm)	93 of 100
No. of grid cells (35 cm)	83 of 100
No. of grid cells (50 cm)	92 of 100
No. of unique grid groups (20 cm)	78
No. of unique grid groups (35 cm)	80
No. of unique grid groups (50 cm)	84
Average grid group size (20 cm)	1.19
Average grid group size (35 cm)	1.04
Average grid group size (50 cm)	1.10
No. of place cells	100 of 100
No. of unique place groups	56
Average place group size	1.79

**Figure 3:**
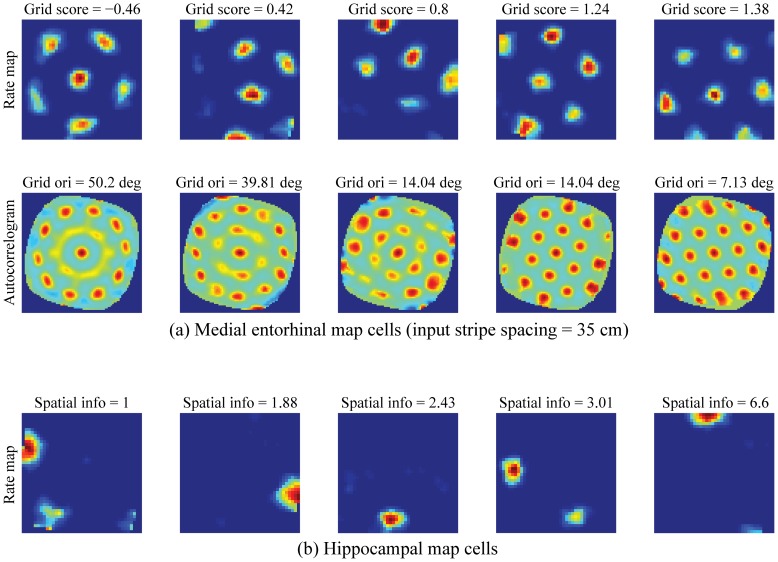
Spatial responses of learned entorhinal cells. (a) Spatial rate maps and autocorrelograms of representative learned entorhinal cells corresponding to the stripe spacing of 35 cm (ranging from lowest to highest grid score). For each of these entorhinal cells, grid score and grid orientation are indicated on top of corresponding rate maps and autocorrelograms, respectively. For example, the values in the rightmost column of panel (a) correspond to grid score of 1.38 and grid orientation of 7.13°. (b) Spatial rate maps of representative learned hippocampal cells (ranging from lowest to highest spatial information) in the last trial. For each of these hippocampal cells, spatial information is indicated on top of corresponding rate map. Color coding from blue (min.) to red (max.) is used in each panel.

Figure 3 presents the spatial responses of five representative learned grid and place cells in the last learning trial. Spatial autocorrelograms of the rate maps are also shown for the grid cells, which in this case were learned from a stripe spacing of 35 cm. These grid and place cells were selected based on uniform sampling of the population distributions of grid score (ranging from −0.46 to 1.38) and spatial information (ranging from 1 to 6.6), respectively. Note the distributed spatial phases of the learned fields at either level in the model hierarchy; namely, the spatially offset firing fields of entorhinal map cells (Figure 3a) and hippocampal map cells (Figure 3b).

**Figure 4:**
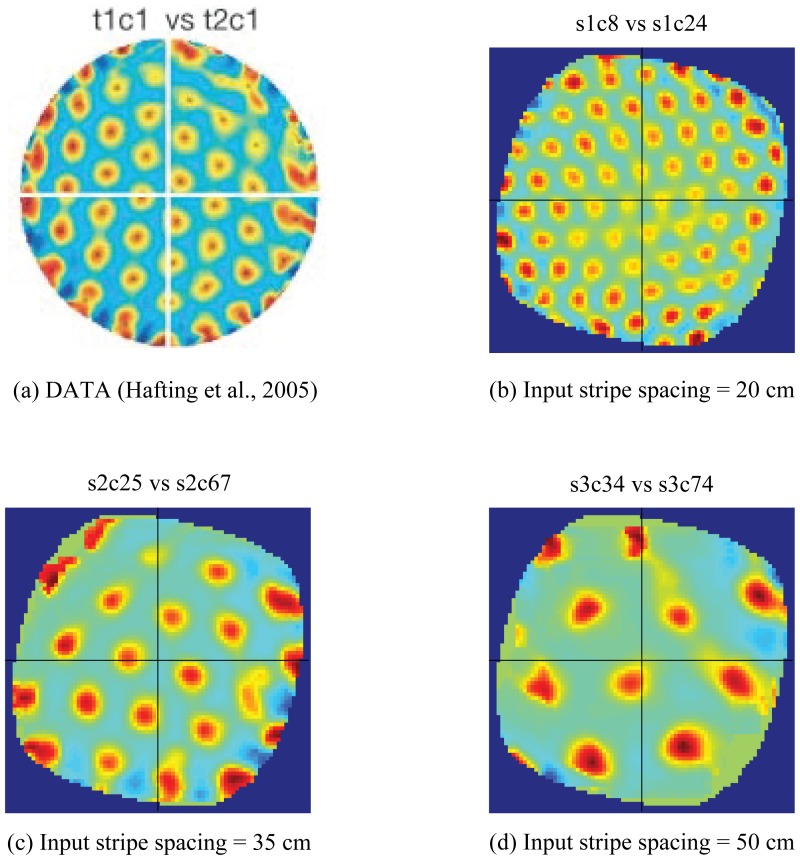
Distributed spatial encoding of learned grid cells. Data (a) and model simulations (b-c) regarding the distributed spatial encoding of grid cells. (a) Cross-correlogram of rate maps of two anatomically nearby grid cells recorded from the rat MEC. (b) Cross-correlogram of rate maps from the last trial of two randomly selected model grid cells (cell #8, cell #24) corresponding to input stripe spacing of 20 cm, similar to (a). (c) Same as in (b) but for input stripe spacing of 35 cm. (d) Same as in (b) but for input stripe spacing of 50 cm. Color coding from blue (−1) to red (1) is used in each panel. [Data reprinted with permission from [8].].

Figure 4 summarizes the distributed spatial encoding by the learned grid cells in the last trial. The firing fields of any two grid cells with the same spacing are formally defined to have different spatial phases if the cross-correlogram of their rate maps does not yield a local maximum at the origin. Moreover, the cross-correlogram exhibits a hexagonal grid pattern if the grid fields of the two cells share nearly the same orientation. In this regard, model simulation results shown in Figures 4b-d, for each of the three spatial scales, closely match characteristic data from grid cells in the adult rat MEC [8] shown in Figure 4a.

**Figure 5:**
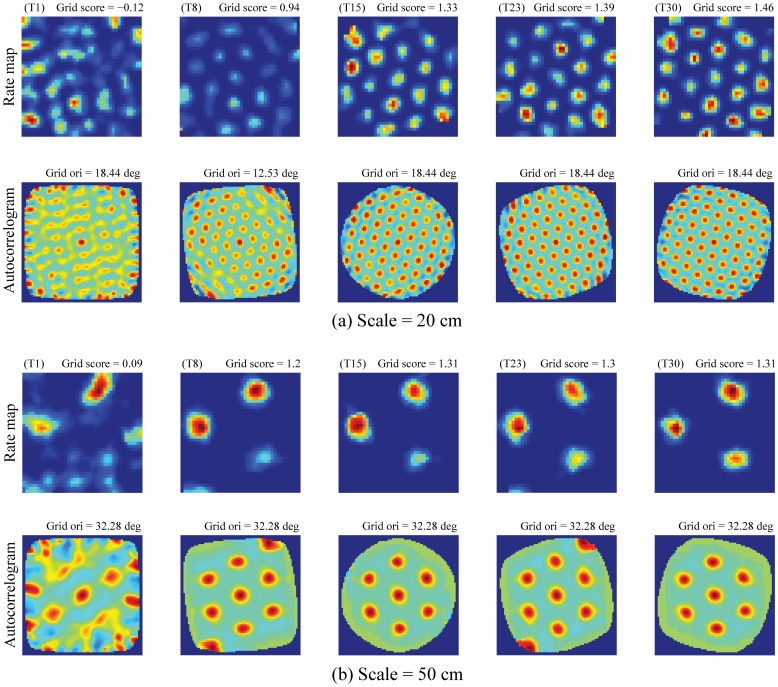
Gradual development of learned grid cells. Evolution of grid firing fields, evident in the rate map and the corresponding autocorrelogram, across learning trials for model grid cells with the highest grid score in the last trial for two of the three input stripe spacings: (a) 20 cm and (b) 50 cm. Note trial number and grid score on top of each rate map, and grid orientation on top of the corresponding autocorrelogram. For example, the values on top of the rate map and autocorrelogram in the first column of panel (a) correspond to first trial (T1), grid score of −0.12, and grid orientation of 18.44°. Color coding from blue (min.) to red (max.) is used for each rate map, and from blue (−1) to red (1) for each autocorrelogram.

Figure 5 shows the gradual evolution of grid firing fields across trials for two entorhinal map cells with the highest grid score in the last trial, one corresponding to the input stripe spacing of 20 cm (Figure 5a) and the other to that of 50 cm (Figure 5b). Comparing the rate maps or autocorrelograms in any trial for these two cells, it can be seen that both the grid field width and spacing increase with the spatial scale of input stripe cells. The time course of hexagonal grid emergence for a given entorhinal cell depends on the pattern of its pre-development weights from stripe cells, the recurrent competitive dynamics within its local entorhinal map, and the amount of time spent by the animat in various regions across space during initial exploration.

**Figure 6:**
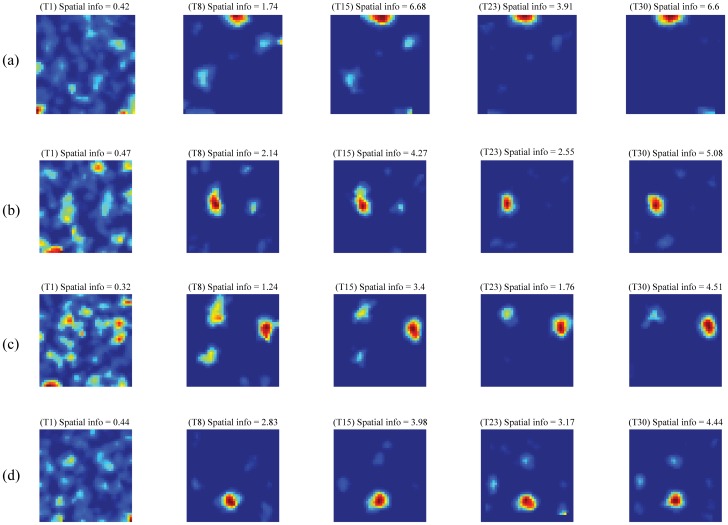
Gradual development of learned place cells. Evolution of spatial firing fields, evident in the rate map, across learning trials for four representative model place cells (a–d). Note trial number and spatial information on top of each rate map. For example, the values on top of the first rate map of panel (a) correspond to first trial (T1) and spatial information of 0.42. Color coding from blue (min.) to red (max.) is used in each panel.

Figure 6 presents the gradual evolution of spatial firing fields across trials for four representative hippocampal map cells. As for entorhinal cells, the time course of place field emergence for a given hippocampal cell depends on the pattern of pre-development weights from its input cells (namely, the entorhinal cells), the recurrent competitive dynamics within the hippocampal map, and the rate at which spatial firing fields of entorhinal cells are incrementally learned. In rat pups, the development of some place cells precedes that of grid cells [34], [35]. These early place cells could result, for example, from learning in response to environmental inputs, such as geometric boundaries and visual landmarks, whose processing may develop sufficiently before that of path integration-based inputs, but they would not be able to represent the large spaces as place cells learned from grid cells. As entorhinal cells mature into those with grid firing fields, downstream hippocampal cells, including those that already have developed some degree of selectivity for different places, are proposed to benefit from integrating these emerging processed spatial signals to enhance the information about the animal's position that their firing carries.

### Development of multimodal place fields

**Figure 7:**
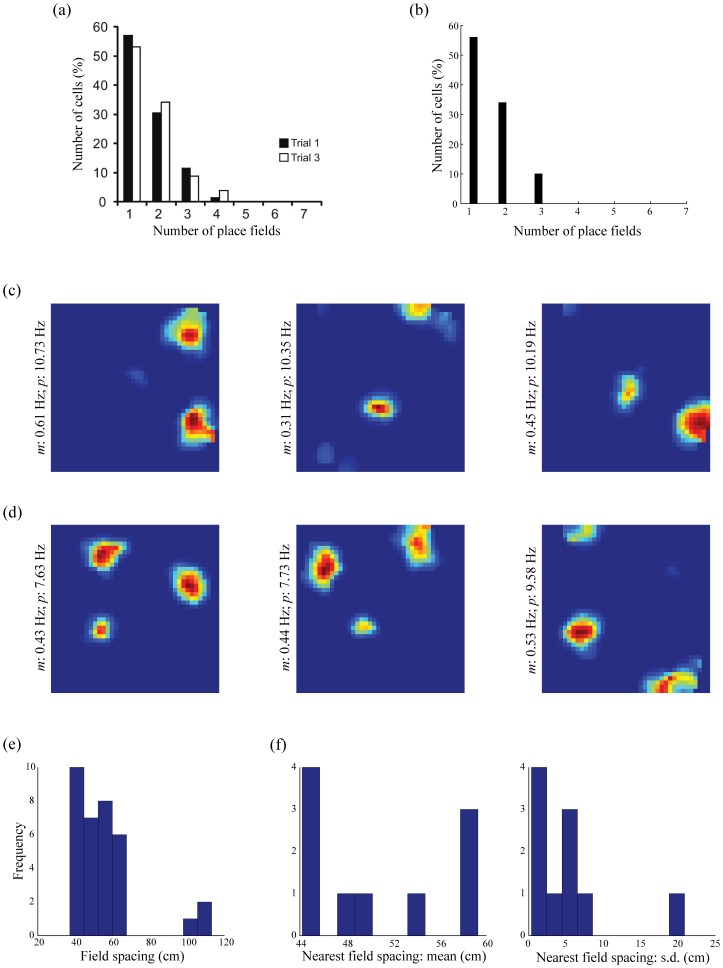
Multimodal firing fields of place cells in large spaces. (a) Data showing a histogram of the number of place fields, in a circular box with a diameter of 200 cm, for dorsal cells in proximal CA1 [6]. (b) Corresponding model simulations for the number of learned place fields, in a square box of 100 cm×100 cm. (c) Smoothed rate maps in the last trial of three representative model place cells expressing two place fields. (d) Smoothed rate maps in the last trial of three representative model place cells expressing three place fields. Note mean (*m*) and peak (*p*) firing rates of the cells along the left side of the corresponding rate maps. (e) Histogram of the field spacing for the model place cells with two place fields. (f) Histograms of the mean and standard deviation of the nearest field spacing for the model place cells with three place fields. [Data reprinted with permission from [6].].

Figure 7 regards the emergence of multimodal place fields (Data: Figure 7a; Model: Figure 7b). A subset of the hippocampal cells do learn more than one place field in the 100 cm×100 cm square box, consistent with data that place cells can have multiple firing fields in larger environments ([5]: 150 cm×140 cm rectangular box; [6]: 200 cm wide circular box; [7]: 180 cm×140 cm rectangular box). In particular, 34% of the hippocampal cells develop with two fields, and 10% with three fields. Figure 7c presents the spatial responses in the last trial of three representative learned place cells with two fields, and Figure 7d similarly presents examples of three fields. Figures 7e and 7f summarize the distribution of the inter-field spacings for all hippocampal map cells with two fields and three fields, respectively. The distribution of standard deviation of nearest field spacings across hippocampal cells with three fields, shown in Figure 7f, reveals that the individual fields are not arranged across space with any particular periodicity, in conformity with similar observations in the pertinent experimental studies [5], [7].

### Learned weights from stripe cells to grid cells

**Figure 8:**
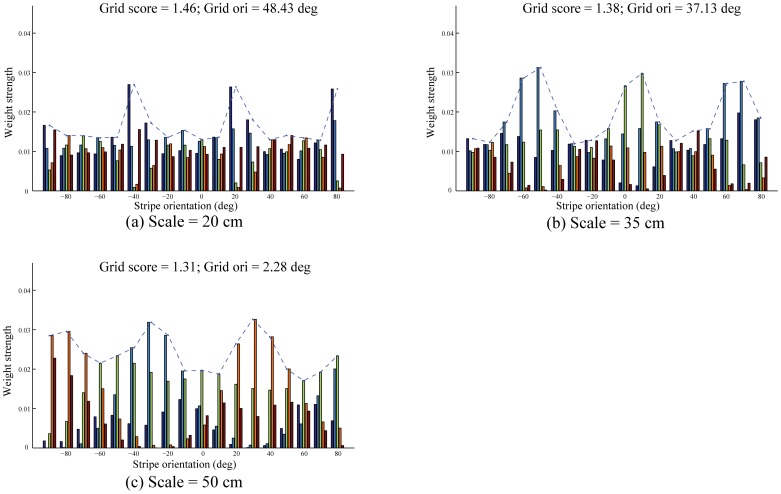
Tuned synaptic weights of learned grid cells. Distribution of adapted weights from stripe cells, grouped by direction, to the model grid cell with the highest grid score in the last trial for each input stripe spacing: (a) 20 cm, (b) 35 cm, and (c) 50 cm. The different colored bars represent different spatial phases of the stripe cells. The dashed line in each panel traces the maximal weights from the various directional groups of stripe cells. Note corresponding grid score and grid orientation on top of each panel.

**Figure 9:**
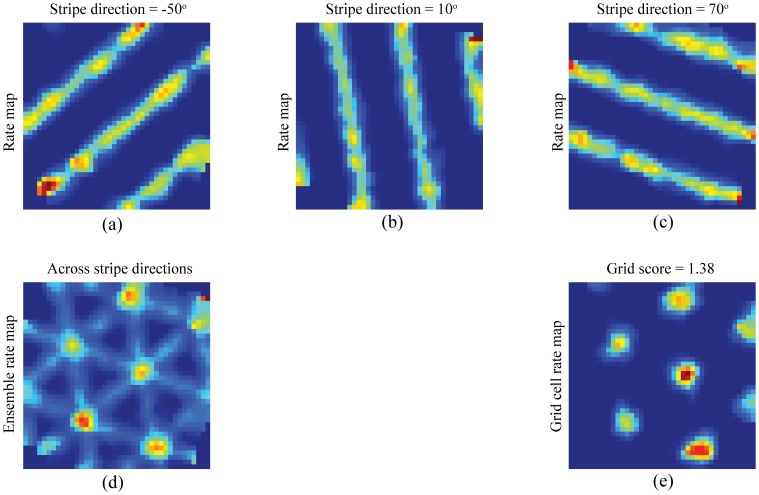
Stripe cell bases of a learned grid cell's receptive fields. Smoothed rate maps in the last trial of (a–c) three stripe cells with a spacing of 35 cm and across directions separated by 60° that project maximally to the model grid cell with the highest grid score in the corresponding entorhinal population. Panel (d) shows the ensemble smoothed rate map of these cells, and panel (e) shows the smoothed rate map of the grid cell under consideration. Color coding from blue (min.) to red (max.) is used in each panel.

Figure 8 shows the bottom-up learned weights from stripe cells to model grid cells with the highest grid score in the three entorhinal maps, for input stripe spacings of 20 cm (Figure 8a), 35 cm (Figure 8b), and 50 cm (Figure 8c), at the end of the last trial. The bars representing weights are grouped by direction with the different colors coding the five spatial phases in each group. These results illustrate that learned grid cells become tuned to selectively respond to coactivations of stripe cells whose preferred directions differ from each other by 60°. In particular, the grid score for a given entorhinal cell correlates with how close the average separation between the local peaks in the distribution of maximal weights from various directional groups is to 60°. For example, these local peaks for the cell shown in Figure 8b, which has a grid score of 1.38, have preferred directions of −50°, 10°, and 70°, which differ from each other by 60°. Figure 9 presents the spatial rate maps in the last trial of stripe cells that correspond to these local peaks, and how their combined rate map accounts for the grid cell's hexagonal grid firing fields. The grid orientation can also be extracted from the set of learned weights from stripe cells. In particular, given the 10° resolution in direction preferences of stripe cells, the grid orientation can be predicted with a 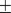
5° margin of error as the direction midway between the above defined local peaks that lies in the range between 0° and 60°. For example, the grid orientation for the cell shown in Figure 8a, namely 48.43°, is near midway between the local peaks at 20° and 80°.

### Learned projections from grid cells to place cells

**Figure 10:**
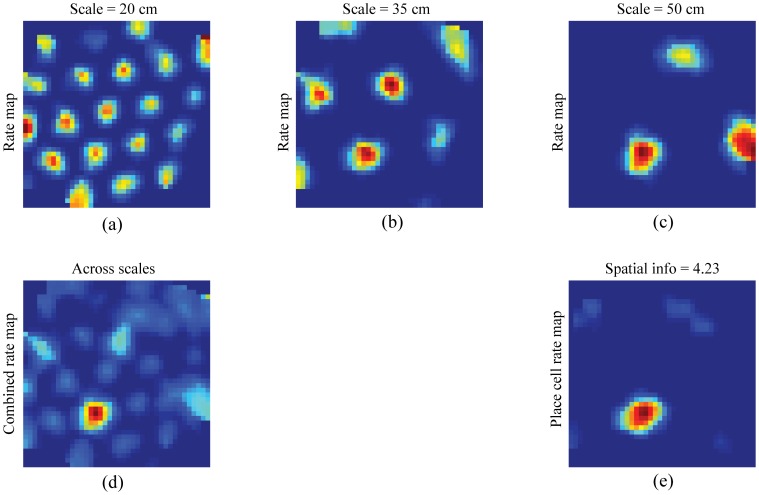
Grid cell bases of a learned unimodal place field. Smoothed rate maps in the last trial of learned grid cells with maximal weights to a representative learned place cell with a *unimodal* place field, for each input stripe spacing separately (a–c) and across spatial scales (d), and of the place cell (e). Color coding from blue (min.) to red (max.) is used in each panel.

**Figure 11:**
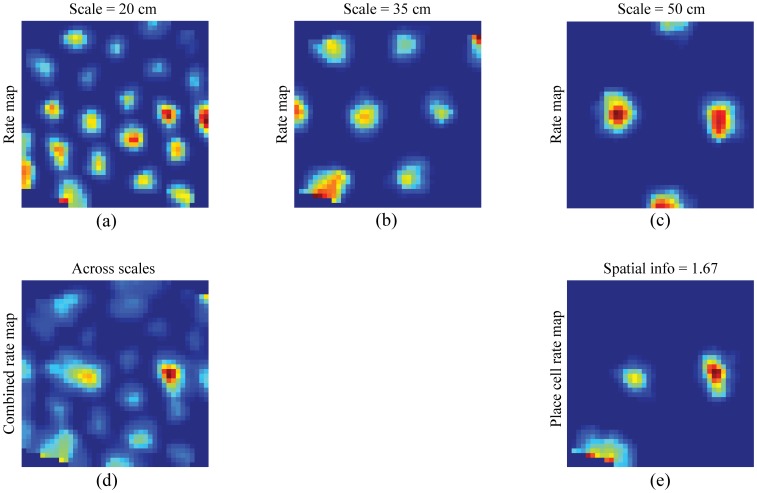
Grid cell bases of multimodal fields of a learned place cell. Smoothed rate maps in the last trial of learned grid cells with maximal weights to a representative learned place cell with *multimodal* place fields, for each input stripe spacing separately (a–c) and across spatial scales (d), and of the place cell (e). Color coding from blue (min.) to red (max.) is used in each panel.

Figure 10 shows the spatial rate maps in the last trial of learned grid cells from each of the three entorhinal maps, for the input stripe spacings of 20 cm (Figure 10a), 35 cm (Figure 10b), and 50 cm (Figure 10c), with maximal weights to one of the 56% of model place cells with single place fields, and how their combined rate map (Figure 10d) highlights the spatial region where the learned grid fields are in phase to account for the place cell's unimodal firing field (Figure 10e). Similarly, Figure 11 shows the spatial rate maps in the last trial of learned grid cells from each of the three entorhinal maps, for the input stripe spacings of 20 cm (Figure 11a), 35 cm (Figure 11b), and 50 cm (Figure 11c), with maximal weights to one of the 10% of model place cells with three place fields, and how their combined rate map (Figure 11d) highlights the three spatial regions where the learned grid fields overlap sufficiently enough to support the place cell's multimodal firing fields (Figure 11e). Multiple place fields for a model place cell can be understood as instances where the activity-dependent competitive selection among entorhinal projections of partial co-activations is sustained. Indeed the average peak rate of place cells with single fields in the last trial is 14.71
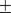
0.3 Hz (mean
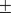
s.e.m.), while that of place cells with multiple fields is 11.37
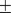
0.45 Hz (right-tailed two-sample t-test: 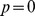). While the mechanisms by which a particular ensemble of place cells are recruited to participate in the representation of a given environment are not clear, our model makes the proposal that if a fixed set of hippocampal cells were to encode space in ever larger environments, there will be greater number of opportunities for partial co-activations of entorhinal inputs to survive the competitive process in causing the firing of hippocampal cells in additional places.

### Net occupancy map and place cell learning

**Figure 12:**
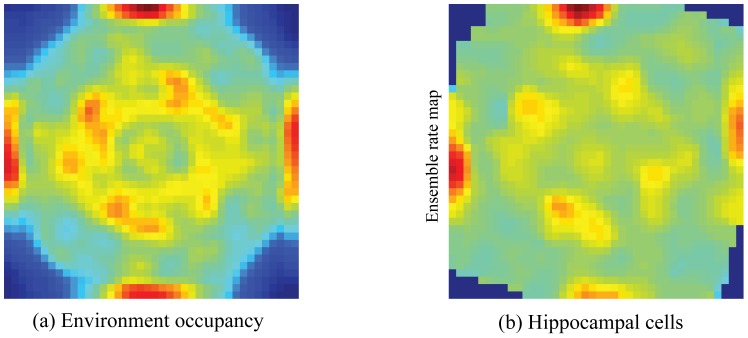
Spatial experience-dependent learning. (a) Environment occupancy map based on the trajectories traveled across the learning trials, and (b) ensemble rate map of all model hippocampal cells in the last trial. Color coding from blue (min.) to red (max.) is used in either panel.

Figure 12 demonstrates that the various learned place fields of hippocampal cells can together encode the dynamic spatial position of the animat in the environment. The net occupancy map, which is obtained by tracking the amount of time spent by the animat in each spatial bin of the environment across all trials, correlates strongly with the ensemble rate map in the last trial of all hippocampal cells (linear correlation: 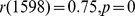), thereby showing that the learned hippocampal code represents various spatial regions depending on the total amount of time spent in them.

### Grid cell development in juvenile rats

**Figure 13:**
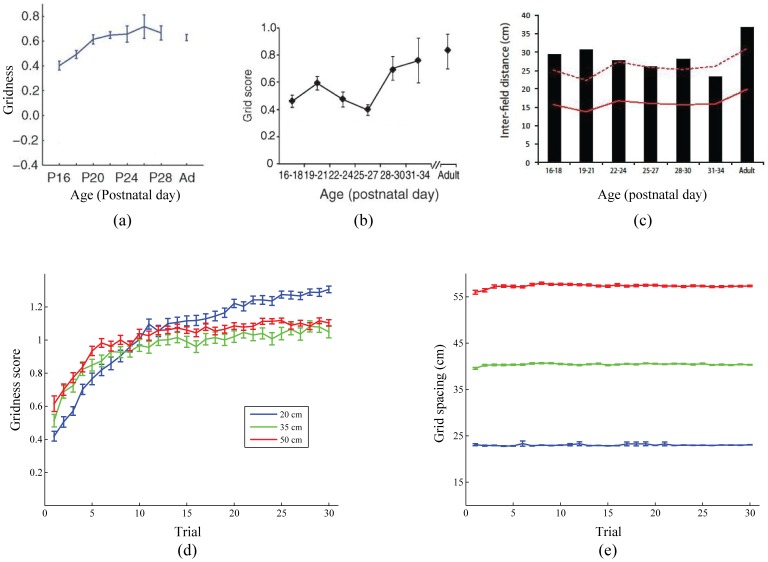
Grid cell development in juvenile rats. (a–c) Data from juvenile rats and (d,e) model simulations regarding the changes in grid cell properties, namely grid score (a: [35]; b: [34]; d: Model) and grid spacing (c: [34]; e: Model), during the postnatal development period. Panels (d) and (e) show simulation results for each input stripe spacing; see legend in panel (d). The error bars correspond to standard error of mean. [Data reprinted with permission from [34], [35].].

Figure 13 shows that the model can replicate data from juvenile rats regarding the development of entorhinal grid cells during postnatal weeks three and four, as two-dimensional space is explored and experienced for the first time [34], [35]. In particular, the model simulates how the average grid score of emerging grid cells gradually improves with learning trial (input stripe spacing of 20 cm: 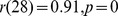; 35 cm: 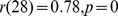; 50 cm: 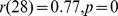), while the average grid spacing does not change significantly (Data: Figures 13a, 13b, and 13c; Model: Figures 13d and 13e). Both are explained together as a reflection of how inputs from stripe cells with the same spacing are gradually modified, across direction preferences and spatial phases.

### Place cell development in juvenile rats

**Figure 14:**
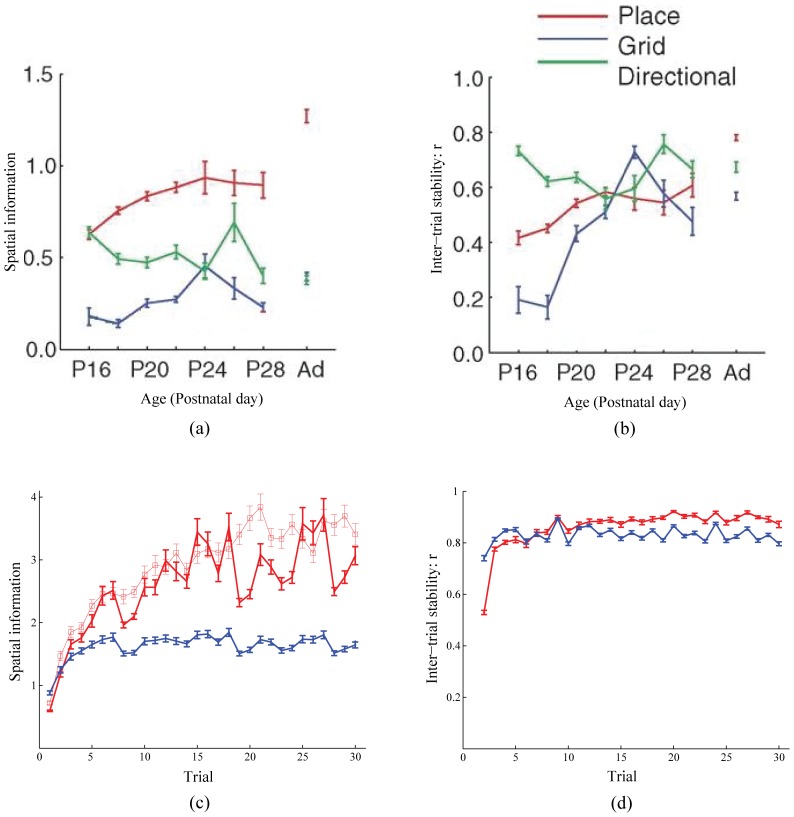
Place cell development in juvenile rats. (a,b) Data from juvenile rats [35] and (c,d) model simulations regarding the changes in place cell properties, namely (a,c) spatial information and (b,d) inter-trial stability, during the postnatal development period. The legend for all panels is in (b). The two red curves, one with dots and the other with squares, correspond to simulations of place cell spatial information during development in response to spatial experience in a 100 cm×100 cm square box and a 100 cm wide circular box, respectively; see **Simulation settings** section for how realistic trajectories for the different trials were generated. The panels also show how corresponding grid cell properties change through rat age/experience (a,b) and learning trials (c,d), respectively. The results for model grid cells shown in (c) and (d) are averaged across the three input stripe spacings. The error bars correspond to standard error of mean. [Data reprinted with permission from [35].].

Figure 14 shows that the model can also account for the data about place cell development in the juvenile rat brain [35]. In particular, the model simulates how the average spatial information of emerging place cells tends to improves with learning trial (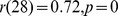), while that of grid cells does not increase as much and is relatively lower (Data: Figure 14a; Model: Figure 14c). While the former reflects gradual self-organization of inputs from entorhinal cells, the latter is the result of multimodal firing fields that grid cells learn. The model also qualitatively simulates the small gradual improvement in the inter-trial stability for place cells during the development period (Data: Figure 14b; Model: Figure 14d [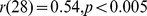]), which results from the gradual stabilization of the weights of projections from developing entorhinal cells.

Though model place cells develop gradually, it can be noticed that their average spatial information content sometimes exhibits marked fluctuations from trial to trial (Figure 14c: red curve with dots). This is due to the particular set of navigational trajectories that were used for the simulation. It may be recalled how a realistic rat trajectory in a *square* box of 100 cm×100 cm (data: [15]) was rotated about the midpoint (origin), which is also the starting position, by random angles to generate the different trajectories. As each new trajectory was bounded by the walls of the box, the animat would spend proportionally more time at particular segments along the four walls depending on the rotation angle. This allowed for potentially wide variations in the time spent by the animat in the various place fields along the walls between the trials. Note that spatial information is defined by 
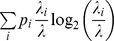
, where 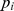 is the proportion of total time spent in a given spatial bin 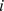 (or, the probability of occupying the bin), 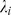 is the firing rate of the cell in bin 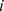, and 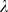 is the mean firing rate across all bins [52]. Given this, other things being equal, the spatial information of a place cell is sensitive to 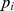
's that correspond to its firing positions. To test this intuition, our model was rerun with a new set of novel trajectories based on a realistic rat trajectory in a 100 cm wide *circular* box (data: [15]). As expected, place cells in this case show a steadier improvement in their spatial information content across the trials; see red curve with squares in Figure 14c.

### Theta modulation

A subset of learned entorhinal and hippocampal cells in the model exhibit theta band modulation [34], [35] as another emergent property of network dynamics, even though model design and parameter values were not geared towards achieving such a temporal coding property. In particular, 62.37% of grid cells for the input stripe spacing of 20 cm (58/93), 24.1% for 35 cm (20/83), and 8.7% for 50 cm (8/92); and 11% of place cells (11/100) are theta-modulated in the last trial; i.e., the mean power within 1 Hz of the peak that is in the theta band (4–12 Hz) of the spike train power spectrum is at least five times greater than the mean power across the 0–125 Hz band [34]. The peak frequency is 9.64
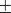
0.063 Hz (mean
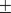
s.e.m.) for theta-modulated grid cells corresponding to input stripe spacing of 20 cm, 10.89
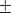
0.16 Hz for 35 cm, and 11.06
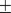
0.15 Hz for 50 cm; and 10.7
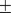
0.24 Hz for theta-modulated place cells. These results are consistent with recent studies showing that theta modulation is not a compulsory signature of the expression of hexagonal grid fields [31], [55], [56].

**Figure 15:**
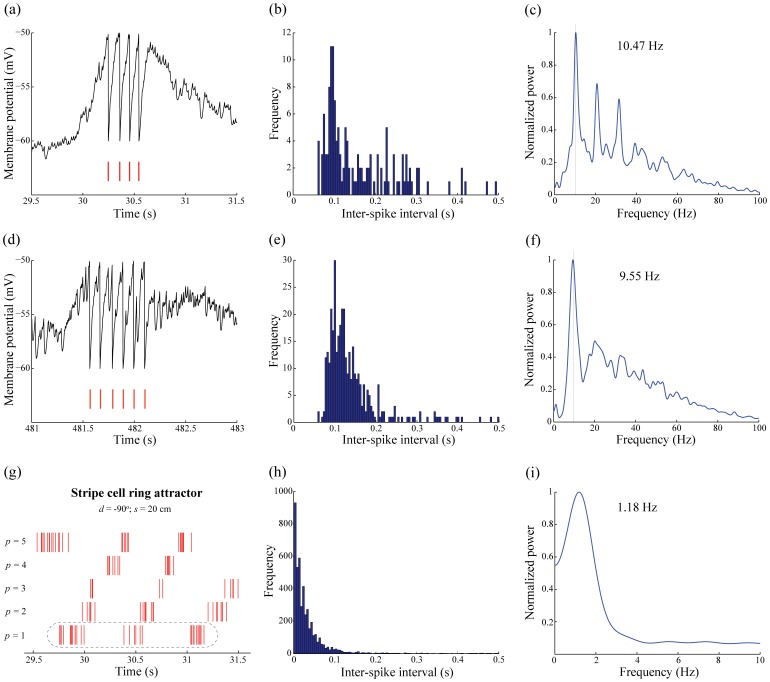
Temporal coding aspects of the various cell types in the model. Representative results are shown for a learned theta-modulated place cell (a–c), a learned theta-modulated grid cell (d–f), and stripe cells (g–i). (a) Membrane potential dynamics of a model place cell in the last trial over a duration of 2 s, with the spiking events highlighted in red. (b) Histogram of the inter-spike intervals (ISIs) for the cell depicted in (a) over the course of the entire last trial (∼9.98 min). (c) Normalized power spectrum computed from the spike train of the cell depicted in (a) over the course of the entire last trial, whose peak frequency of 10.47 Hz falls within the theta band (4–12 Hz). Along the same lines, panels (d–f) show membrane potential dynamics, ISI histogram, and spike train normalized power spectrum of a model grid cell for the input stripe spacing of 35 cm. (g) Raster plot of spikes from input stripe cells 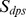 of different spatial phases belonging to a ring attractor with a spatial scale of 20 cm and a preferred direction of −90°, over a duration of 2 s in the last trial. Panels (h) and (i) show the ISI histogram and normalized power spectrum for the stripe cell highlighted in (g) with a dashed round rectangle.

Figures 15a and 15d display representative membrane potential dynamics of a theta-modulated model place cell and grid cell, respectively, in response to traversals through their respective spatial fields. Figure 15 also provides the histograms of inter-spike intervals (ISIs) for these cells (Figures 15b and 15e), which help to account for the intrinsic theta firing frequencies in their corresponding spike train-based power spectra (Figures 15c and 15f). Figure 15g shows typical spiking patterns in a raster plot of input stripe cells of different spatial phases belonging to a ring attractor that integrates linear velocity along a particular direction (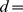
−90°) and spacing (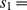
20 cm). Figures 15h and 15i provide the ISI histogram and spike train power spectrum of one of the stripe cells, which highlight the lack of modulation in the theta band. This is true for all the stripe cells in the model. It must be noted, however, they are currently implemented algorithmically as realizations of non-homogenous Poisson processes. The dynamic characterization of stripe cell ring attractors is a topic for future research.

### Implementing sGridPlaceMap in neuromorphic hardware

A principled way to achieve unprecedented levels of natural intelligence in future mobile robots is to design their controllers to emulate the as-yet unrivaled abilities for learning flexible, adaptive behaviors that are exhibited by advanced biological brains in response to unexpected challenges in ever-changing environments. It has been broadly acknowledged that Moore's law, which predicted the doubling of transistor density on computer chips every two years, and corresponding speed-ups in chip performance, will breakdown within the next 10 years due to physical limitations. In particular, at very high densities, the resulting nano-scale chips will be noisy and unreliable, thereby catastrophically degrading the functioning of digital computers. Denser chips also generate more heat that can cause meltdown. One biologically-inspired way to generate less heat is to use temporally discrete signals, or spikes, for information transmission, and at lower rates if possible. Further, the processing power of computers is limited by the finite bandwidth of communication between the physically-separated central processing unit and main memory. This *von Neumann bottleneck* can become increasingly problematic with very high density chips.

In sharp contrast to the serial architecture employed in present-day computing machines, biological brains have a massively parallel architecture in which learning and memory processes are distributed across local circuits that are composed of noisy spiking neurons. Despite a high density of neurons and their connections (one million neurons and ten billion synapses per sq. cm.), each human brain consumes just about 20 W of power. This power budget contrasts dramatically with that required (∼300,000 times more) to run the most advanced supercomputer in the world; namely, the Blue Gene/Q at the Lawrence Livermore National Laboratory in Livermore, CA. Moreover, such advanced supercomputers occupy a lot of physical space, and need to be explicitly programmed for each specific task that they are supposed to perform. Aggressive efforts are currently underway across the world to develop a fundamentally new class of computer chips that closely mirror biological brains to herald the arrival of a transformative new technological field of natural intelligence. With respect to sGridPlaceMap model computations, the spiking competitive instar learning law described in Equations 9 and 16 can be rewritten in a form that facilitates better, more local implementation in neuromorphic hardware as follows:



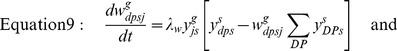
(19)




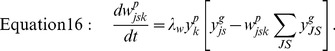
(20)


This form reveals a single inhibitory term (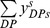 in Equation 19, and 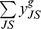 in Equation 20), which can be computed at a non-specific inhibitory interneuron that broadcasts the same value to all bottom-up synapses.

**Figure 16:**
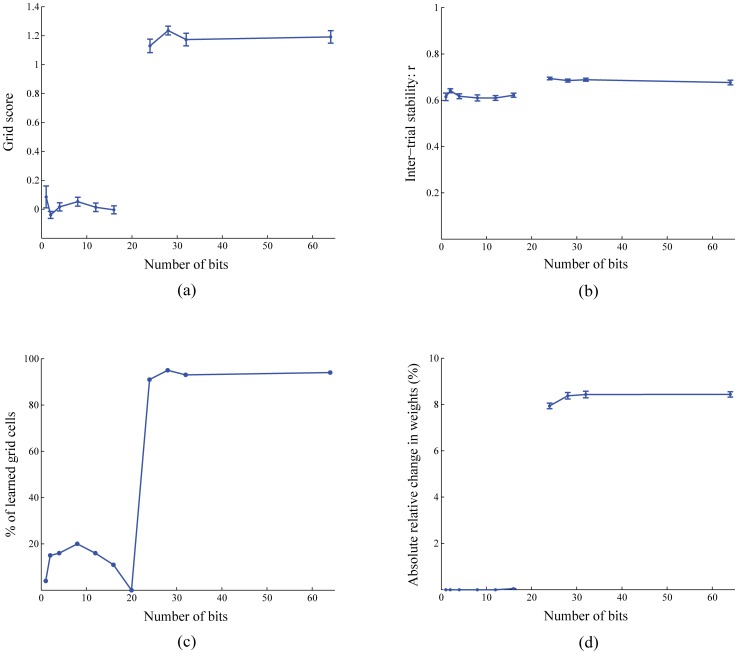
Towards neuromorphic implementation of sGridPlaceMap model. Quality of spiking grid cell learning, in response to stripe cells with a spacing of 20 cm, as a function of number of bits available to store synaptic weights with regard to (a) grid score, (b) inter-trial stability, and (c) percentage of learned grid cells at the end of 10 trials. Panel (d) shows the absolute relative change in weights from input stripe cells, as a percentage, through the tenth trial as a function of number of bits.

Also, the minimum number of bits to represent synaptic weights that can support the learning of spiking grid cells was determined. New simulations of grid cell learning, in response to stripe cells with a spacing of 20 cm, were run with synaptic weights at each time step being rounded off to one of a finite number of discrete levels 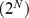 between 0 and 1, which are dependent on the available number of bits 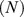. Different values of 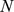 were tested; namely, 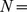
1, 2, 4, 8, 12, 16, 20, 24, 28, 32, and 64. The initial weights were sampled from a uniform distribution between 0 and 1. Quality of learning for each map cell was assessed by computing the standard grid score and inter-trial stability at the end of 10 learning trials. Results shown in Figure 16 reveal that in order for the slow weight changes at each time step to be registered, at least 20 bits are needed. And for non-trivial grid cells to be learned, at least 21 bits are needed. Interestingly, more than 21 bits do not seem to bring any additional benefit with regard to grid score, inter-trial stability, and proportion of learned grid cells. These results help to differentiate neuromorphic approaches employing artificial synaptic components that are capable of multilevel storage (e.g., [57]) from those that only allow binary storage (e.g., [58]), for the purpose of matching the hardware and software specifications and constraints of the brain.

## Discussion

Understanding how the entorhinal-hippocampal system learns grid and place cells is needed as a foundation for developing a comprehensive theory of how spatial cognition works in humans and higher animals, as well as for developing controllers of autonomous adaptive mobile robots that use only locally available signals to navigate to remembered locations of valued goal objects. The current article builds upon insights gained from our prior rate-based modeling of grid and place cell development [29] to simulate how spiking hippocampal place cells can be learned based on most frequent and energetic co-excitatory inputs from spiking medial entorhinal cells that are concurrently self-organizing into grid cells in response to most frequent and energetic co-excitatory inputs from spiking stripe cells during navigation along realistic trajectories. This stripe-to-grid-to-place adaptive transformation of linear velocity estimates, as a young animal freely explores open space beyond its nest for the first time (P15-P28), allows the hippocampus to greatly expand the scale of its representation of space, thereby enabling efficient (around P28: [34]) and behaviorally-useful navigation. The current article also demonstrates the appearance of theta band modulation, thereby paving a way for mechanistically studying temporal coding in the entorhinal-hippocampal system, and the emergence of multimodal place fields as emergent effects of the model dynamics.

### Predictions about spatial learning in piecewise linear environments

The sGridPlaceMap model makes testable experimental predictions. For example, rats that have early spatial experience in only piecewise linear underground tunnels, as happens in nature, are predicted to learn a fewer proportion of hexagonal grid cells than rats that navigate in open fields. This is because the resultant sparser coverage of two-dimensional space allows only a subset of hexagonal grid exemplars to be experienced by the would-be grid cells. Note that for a grid exemplar to be learned, the animal, or animat, needs to traverse through at least three places that are part of the grid template. Also, the grid cells that may develop during piecewise linear navigation are predicted to have a lower hexagonal gridness quality. This is because in a one-dimensional environment, such as a linear track, sets of co-active stripe cells that are most frequent and energetic turn out not to be the ones that generate hexagonal grid structures, but those that comprise two stripe cells whose preferred directions differ by 90° with the linear space coincident with a spatial field of one of them.

### Theta phase precession in grid cells and place cells

The phenomenon of theta phase precession is exhibited by place cells in hippocampal areas CA1 and CA3 [59], and grid cells in layer II of MEC [60]. Phase precession occurs when the phase of the theta rhythm at which a space-encoding cell fires tends to gradually move to earlier values in subsequent theta cycles during traversal of the animal through the cell's spatial receptive field [59]. The theta phase precesses from about 355° coinciding with entry into the spatial field to about 100° during exit, on average across trials and cells. For grid cells, phase precession is seen for movement through each grid field [60] that is independent across fields [61]. For place cells, the rate of phase precession has been shown to increase with running speed [62] and to be greater for smaller place fields [63]. While whether neural information is encoded in the frequency or timing of spikes is still an open question in the field, proponents of temporal coding for spatial navigation rely on analyses that show the amount of spatial information carried by a cell's firing rate is greatly enhanced, and thereby the accuracy of spatial position decoding based on the ensemble code, when firing phase is also considered (place cells: [64]; grid cells: [61]). Existing models of phase precession [53], [65]–[68] assume the local field potential (LFP) signal to be a given. While some researchers propose that the hippocampal theta rhythm arises from the theta pacemaker cells in the medial septum (e.g., [69]–[71]), others invoke local network interactions (e.g., [72], [73]). Buzsaki and colleagues have presented a computational model to demonstrate both the network theta rhythm and its slower frequency compared to phase precessing place cells may emerge naturally as the population output of different place cells, with offset place fields, that fire inherently at frequencies faster than that of the theta rhythm [74]. Along this line, our future work will include enhancing the sGridPlaceMap model with a way to explicitly model the LFP signal to contribute towards a more complete mechanistic explanation of theta phase precession.

### Scale selection problem

How multiple-scale spatial representations across the dorsoventral axis in layer II of MEC [16], [17] are self-organized is an important question because multiple spatial scales of grid cells are needed for the ensemble of hippocampal pyramidal cells to learn to function as a self-localization system. This is also a difficult problem because, before development begins, grid cells may receive inputs from stripe cells of several spatial scales. The simulations of the current model and those of GRIDSmap [23] and GridPlaceMap [29] assumed that the grid cells of a given spatial scale are learned directly from stripe cells of the corresponding scale. [75] reported a decreasing dorsoventral gradient in the average frequency of subthreshold membrane potential oscillations (MPOs) in response to steady current inputs for MEC layer II stellate cells, while [16] observed decreasing average peak and mean firing rates. [76] found a dorsoventral gradient in the average rate of temporal integration for these cells. In particular, they showed that both the rise and fall times of EPSPs tend to increase along the dorsoventral axis, even though the underlying synaptic currents are the same. Moreover, [77] reported that spike afterhyperpolarization potential (AHP) kinetics also vary systematically, with the AHP decay time constant (and thereby duration) increasing, on average, from the dorsal to the ventral end. In other words, the relative refractory period tends to be shorter for dorsal cells and longer for ventral cells.

Theoretically integrating all these data in a rate-based model, [30] demonstrated that the gradient in grid spatial scales can be learned by SOM cells that respond with monotonically decreasing response rates along the dorsoventral extent of MEC. It is the variable-rate refractory dynamics that directly help to select the spatial scale of stripe cells to which the grid cells learn to respond. Said in another way, dorsal cells with shorter refractory periods prefer input coactivations that reoccur, on average, with a smaller temporal interval for most frequent and energetic activation, and ventral cells prefer those that reoccur with a larger temporal interval.

This gradient of cell response rates also enabled the model to simulate the observed gradients in MPO frequencies, firing rates, and refractory periods as emergent properties of SOM learning mechanisms. Among the several input variations that were simulated, it was found that only a response rate gradient combined with input stripe cells that have normalized receptive fields across scales can provide an account for the dorsoventral variations in all above mentioned spatial and temporal properties of entorhinal grid cells.

These results show that the anatomical gradient in the temporal frequency of intrinsic oscillations [75], [78] can occur in the absence of an oscillatory interference-based mechanism for grid cell firing (compare with [68], [75], [79], etc.). Consistently, an investigation of grid cells in mice with knockout of subunit 1 in the hyperpolarization-activated cyclic nucleotide-gated (HCN1) channels [80] concluded that the development of the grid scale spectrum is more dependent on the gradient in the rate of temporal integration, which occurs due to dorsal-to-ventral decreases in the amplitudes of leak K^+^ and HCN1 channel conductances ([76]: mouse), than the gradient in resonant properties such as MPO frequency, due to dorsal-to-ventral increases in the time constants of the HCN1 channel conductance ([81]: rat).

The spiking grid and place model developed in this article makes it feasible to ascertain more directly the relative contributions of and interactions among different synaptic currents in setting up the spatial scale topography of grid cells, and to probe them further computationally; namely, the AHP, leak K^+^, and hyperpolarization-activated cation (*I*_h_) currents mentioned above, and the m-current [82], among others. For instance, the membrane potential recovery from afterhyperpolarization in MEC layer II stellate cells is known to be quickened in proportion to the action of *I*_h_ [83]. Also, the fast and slow-medium currents that determine AHP in stellate cells [84] may have potentially different effects [85]. Moreover, the role of the persistent sodium (NaP) current, whose interplay with *I*_h_ is known to generate subthreshold MPOs [86], is not fully clear.
